# Supplementary material for: Parental vaccine hesitancy and acceptance of a COVID-19 vaccine: An internet-based survey in the US and five Asian countries
Source: PLOS Glob Public Health. 2024 Feb 28;4(2):e0002961. doi: 10.1371/journal.pgph.0002961 (PMC10901326; doi:10.1371/journal.pgph.0002961)
Supplement: S1 Checklist — (DOCX) [file pgph.0002961.s001.docx]

STROBE Statement—checklist of items that should be included in reports of observational studies

|  | Item No. | Recommendation | Page  No. | Relevant text from manuscript |
| --- | --- | --- | --- | --- |
| **Title and abstract** | 1 | (*a*) Indicate the study’s design with a commonly used term in the title or the abstract | 2 | We analyzed data from an opt-in, internet-based cross-sectional study |
|  |  | (*b*) Provide in the abstract an informative and balanced summary of what was done and what was found | 2 | Materials and Methods: We analyzed data from an opt-in, internet-based cross-sectional study (n=23,940). Parents were asked about their acceptance of a COVID-19 vaccine for their children, and if they would accept the vaccine with different risk and effectiveness profiles for themselves. Poisson regression was used to estimate the relationship between vaccine acceptance for a child and vaccine profile, by country and waves and overall. Survey procedures were also used to estimate the frequency of discordance between parents and their children regarding vaccine acceptance, considering vaccine hesitancy and education level.  Results: Over time, COVID-19 vaccine acceptance for children decreased in the United States and Taiwan, while it increased in Indonesia and Malaysia. The population attributable fraction of non-vaccinated children with vaccine-hesitant parents was highest in Taiwan (36.1%, March 2021) and in the United States (26.3%, February 2021). Vaccine risk and effectiveness profiles did not consistently affect parent’s acceptance of a COVID-19 vaccine for their children. Overall, parents preferred a safer, more effective vaccine for their children. The United States and Taiwan showed higher discordance in parents’ vaccine preferences for children and themselves compared to other countries such as China, Indonesia, India, and Malaysia |
| Introduction | | | |  |
| Background/rationale | 2 | Explain the scientific background and rationale for the investigation being reported | 3-4 | various, including:  Concerns surrounding vaccine safety have been present since the first smallpox immunization campaigns [9]. Various studies have attempted to examine the factors contributing to parents’ vaccine hesitancy towards COVID-19 vaccination for their children  Currently, there is limited research on the decision-making of parents of children under 18 regarding COVID-19 vaccination, particularly in the context of cross-country comparisons |
| Objectives | 3 | State specific objectives, including any prespecified hypotheses | 4 | Therefore, the objective of this study is to investigate parent’s vaccine behaviors and their decisions related to COVID-19 vaccination for their children in selected countries. |
| Methods | | | |  |
| Study design | 4 | Present key elements of study design early in the paper | 4 | This study used an opt-in, internet-based sample that was recruited through social media and online advertisements by a survey research firm. Cross-sectional, online surveys were conducted in six countries |
| Setting | 5 | Describe the setting, locations, and relevant dates, including periods of recruitment, exposure, follow-up, and data collection | 4 | Cross-sectional, online surveys were conducted in six countries, including the US, China, Indonesia, India, Malaysia, and Taiwan in August 2020, November 2020, March 2021, and June 2021. An additional survey was conducted in China in March 2020 and in the US in June 2020, October 2020, February 2021, and April 2021. |
| Participants | 6 | (*a*) *Cohort study*—Give the eligibility criteria, and the sources and methods of selection of participants. Describe methods of follow-up  *Case-control study*—Give the eligibility criteria, and the sources and methods of case ascertainment and control selection. Give the rationale for the choice of cases and controls  *Cross-sectional study*—Give the eligibility criteria, and the sources and methods of selection of participants | 4 | This study used an opt-in, internet-based sample that was recruited through social media and online advertisements by a survey research firm. Cross-sectional, online surveys were conducted in six countries.  […]  The eligibility criteria included being an adult residing in the country where the data were collected. |
|  |  | (*b*) *Cohort study*—For matched studies, give matching criteria and number of exposed and unexposed  *Case-control study*—For matched studies, give matching criteria and the number of controls per case | NA |  |
| Variables | 7 | Clearly define all outcomes, exposures, predictors, potential confounders, and effect modifiers. Give diagnostic criteria, if applicable | 4 | During the survey, participants were asked if they had children under the age of 18. If they responded in the affirmative, they were then asked a question about their acceptance of a coronavirus vaccine for their children: “Would you accept a coronavirus vaccine for your child?” Participants were also asked whether they had received a coronavirus vaccine, planned to receive a vaccine, or had already been vaccinated. Using these two variables, we created a variable to examine discordant vaccine acceptance between the parent and child (wanting a vaccine for self and child, wanting a vaccine for self but not for child, wanting a vaccine for child but not for self, or not wanting a vaccine for self or child). We also measured adult vaccine hesitancy using the validated 10-item adult Vaccine Hesitancy Scale (aVHS) [11]. The aVHS had a 5-point Likert scale as answer choices, ranging from least hesitant (1) to most hesitant (5). Based on a published standard, we dichotomized this variable into vaccine hesitant and non-vaccine hesitant categories [12]. Finally, to understand how safety and effectiveness of vaccines influence decision-making, parents were asked if they would accept a vaccine with varying levels of safety and effectiveness for themselves (50% effective with a 20% risk of fever; 50% effective with a 5% risk of fever; 95% effective with a 20% risk of fever; or 95% effective with a 5% risk of fever). |
| Data sources/ measurement | 8* | For each variable of interest, give sources of data and details of methods of assessment (measurement). Describe comparability of assessment methods if there is more than one group | 4-5 | We conducted our analyses using SAS Version 9.4 (Cary, North Carolina). Specifically, we used a Poisson regression model to estimate prevalence ratios (PRs) for vaccine acceptance for a child in each country and wave in this study, using the vaccine profile variable. We also used Poisson regression to estimate PRs and 95% confidence intervals (CIs) for the association between vaccine acceptance for a child and the vaccine profile, vaccine hesitancy, education level, and the month of the survey, when all countries were combined. We used survey procedures to estimate the frequency of discordant vaccine views between self and child by vaccine hesitancy and education level, separately for each country in the June 2021 wave.  Given that the data was weighted to be representative of populations in terms of age, gender, and race, survey procedures were used in our analyses. We used weights when we combined the data for all six countries, ensuring that each country was given equal weight. Finally, we used log binomial models and the frequency of non-vaccination of children with vaccine-hesitant parents to estimate the fraction of non-vaccination of children that was related to parental vaccine hesitancy. |
| Bias | 9 | Describe any efforts to address potential sources of bias | N/A | (Obviously our study has biases. We talk about those in the limitations) |
| Study size | 10 | Explain how the study size was arrived at | 4 | For each wave, we attempted to obtain a sample size of 800, in order to estimate an outcome proportion of 50% (a statistically conservative estimate of the population vaccinated), based on a margin of error was 4% and with an alpha of 0.05 and a power of 80%. |

Continued on next page

| Quantitative variables | 11 | Explain how quantitative variables were handled in the analyses. If applicable, describe which groupings were chosen and why | 5 | We also used Poisson regression to estimate PRs and 95% confidence intervals (CIs) for the association between vaccine acceptance for a child and the vaccine profile, vaccine hesitancy, education level, and the month of the survey, when all countries were combined. This analysis used education as a socioeconomic confounder common to all countries’ surveys. |
| --- | --- | --- | --- | --- |
| Statistical methods | 12 | (*a*) Describe all statistical methods, including those used to control for confounding | 5 | This analysis used education as a socioeconomic confounder common to all countries’ surveys. |
|  |  | (*b*) Describe any methods used to examine subgroups and interactions | 5 | we used a Poisson regression model to estimate prevalence ratios (PRs) for vaccine acceptance for a child in each country and wave in this study, using the vaccine profile variable. |
|  |  | (*c*) Explain how missing data were addressed | 5 | Individuals with missing data were excluded from analysis. |
|  |  | (*d*) *Cohort study*—If applicable, explain how loss to follow-up was addressed  *Case-control study*—If applicable, explain how matching of cases and controls was addressed  *Cross-sectional study*—If applicable, describe analytical methods taking account of sampling strategy | 5 | The data were weighted to be representative of national populations in terms of age, gender, and race |
|  |  | (*e*) Describe any sensitivity analyses | N/A |  |
| Results | | | | |
| Participants | 13* | (a) Report numbers of individuals at each stage of study—eg numbers potentially eligible, examined for eligibility, confirmed eligible, included in the study, completing follow-up, and analysed | 5 | The sample size for this analysis was 23,940 participants across all waves and countries. Each wave of data collection included over 630 participants. More information on the sample size, the number of participants who agreed to participate in this study, and the number of participants who completed the study can be found online (https://doi.org/10.6084/m9.figshare.14792058.v3) . |
|  |  | (b) Give reasons for non-participation at each stage | 5 | (same as above) |
|  |  | (c) Consider use of a flow diagram | N/A |  |
| Descriptive data | 14* | (a) Give characteristics of study participants (eg demographic, clinical, social) and information on exposures and potential confounders | N/A | the exposure was randomized |
|  |  | (b) Indicate number of participants with missing data for each variable of interest | 5 | More information on the sample size, the number of participants who agreed to participate in this study, and the number of participants who completed the study can be found online (https://doi.org/10.6084/m9.figshare.14792058.v3) . |
|  |  | (c) *Cohort study*—Summarise follow-up time (eg, average and total amount) |  |  |
| Outcome data | 15* | *Cohort study*—Report numbers of outcome events or summary measures over time |  |  |
|  |  | *Case-control study—*Report numbers in each exposure category, or summary measures of exposure |  |  |
|  |  | *Cross-sectional study—*Report numbers of outcome events or summary measures | 5 | Figure 1 shows the distribution of vaccine acceptance for children by country and wave. In the US, parent’s acceptance of a COVID-19 vaccine for their children ranged from 67% in March 2021 to 90% in June 2020. This indicated an overall decrease in acceptance across all waves. The trend of declining vaccine acceptance over time was also observed in Taiwan, where acceptance ranged from 64% in March 2021 to 79% in August 2020. Conversely, in China and Indonesia, acceptance remained relatively stable around 90% and 86%, respectively, across all waves. India and Malaysia demonstrated an overall increase in vaccine acceptance over time. |
| Main results | 16 | (*a*) Give unadjusted estimates and, if applicable, confounder-adjusted estimates and their precision (eg, 95% confidence interval). Make clear which confounders were adjusted for and why they were included |  | Table 2 shows unadjusted estimates, Table 3 adjusted. |
|  |  | (*b*) Report category boundaries when continuous variables were categorized | N/A |  |
|  |  | (*c*) If relevant, consider translating estimates of relative risk into absolute risk for a meaningful time period | N/A | (we decided to just use prevalence ratios) |

Continued on next page

| Other analyses | 17 | Report other analyses done—eg analyses of subgroups and interactions, and sensitivity analyses | 6-10 |  |
| --- | --- | --- | --- | --- |
| Discussion | | | | |
| Key results | 18 | Summarise key results with reference to study objectives | 12 | In this large cross-sectional study, encompassing six different countries and multiple waves of data, we identified discernible variations in COVID-19 vaccine acceptance for children both within countries over time. Specifically, we found a general decrease in vaccine acceptance for children in the US and Taiwan, while Malaysia and India had an increase in vaccine acceptance. China and Indonesia, in contrast, displayed a stable level of vaccine acceptance. In Malaysia, the initiation of the adult COVID-19 vaccination program in February 2021 [13] led to an increase in parents' confidence in vaccinating their children, rising from 81% in August 2020 to 84% in March 2021 |
| Limitations | 19 | Discuss limitations of the study, taking into account sources of potential bias or imprecision. Discuss both direction and magnitude of any potential bias | 14 | This study used data from an opt-in, internet-based sample, which may introduce bias and limit generalizability to the broader population. However, this sampling approach allowed for efficient and timely data collection, particularly, given the circumstances imposed by the pandemic. It is important to acknowledge that participants were required to have internet access to complete the survey, which may introduce a potential source of bias in the sample. In addition, the reliance on self-reported data may be subject to social desirability bias, potentially affecting the validity of responses. Nevertheless, this study employed consistent survey methods across six countries and multiple waves, enabling meaningful comparisons of results both within and between countries over time. This approach provides valuable insights into cross-country variations and trends related to vaccine attitudes and behaviors, contributing to our understanding of the broader landscape of vaccine hesitancy. |
| Interpretation | 20 | Give a cautious overall interpretation of results considering objectives, limitations, multiplicity of analyses, results from similar studies, and other relevant evidence | 12-14 |  |
| Generalisability | 21 | Discuss the generalisability (external validity) of the study results | 12 | . It is important to acknowledge that participants were required to have internet access to complete the survey, which may introduce a potential source of bias in the sample. In addition, the reliance on self-reported data may be subject to social desirability bias, potentially affecting the validity of responses. |
| Other information | |  | | |
| Funding | 22 | Give the source of funding and the role of the funders for the present study and, if applicable, for the original study on which the present article is based | in application system | This project was supported by an award from the National Science Foundation, Division of Social and Economic Sciences (#2027836). |

*Give information separately for cases and controls in case-control studies and, if applicable, for exposed and unexposed groups in cohort and cross-sectional studies.

**Note:** An Explanation and Elaboration article discusses each checklist item and gives methodological background and published examples of transparent reporting. The STROBE checklist is best used in conjunction with this article (freely available on the Web sites of PLoS Medicine at http://www.plosmedicine.org/, Annals of Internal Medicine at http://www.annals.org/, and Epidemiology at http://www.epidem.com/). Information on the STROBE Initiative is available at www.strobe-statement.org.
