# Supplementary material for: Parental vaccine hesitancy and acceptance of a COVID-19 vaccine: An internet-based survey in the US and five Asian countries
Source: PLOS Glob Public Health. 2024 Feb 28;4(2):e0002961. doi: 10.1371/journal.pgph.0002961 (PMC10901326; doi:10.1371/journal.pgph.0002961)
Supplement: Attachment — Submitted filename: response to reviewers.docx [file pgph.0002961.s003.docx]

**1. Please include a complete copy of PLOS’ questionnaire on inclusivity in global research in your revised manuscript. Our policy for research in this area aims to improve transparency in the reporting of research performed outside of researchers’ own country or community. The policy applies to researchers who have travelled to a different country to conduct research, research with Indigenous populations or their lands, and research on cultural artefacts. The questionnaire can also be requested at the journal’s discretion for any other submissions, even if these conditions are not met. Please find more information on the policy and a link to download a blank copy of the questionnaire here: https://journals.plos.org/globalpublichealth/s/best-practices-in-research-reporting. Please upload a completed version of your questionnaire as Supporting Information when you resubmit your manuscript.**

**Authors’ response:**

-----

**2. Please provide separate figure files in .tif or .eps format.**

**For more information about figure files please see our guidelines:**

**https://journals.plos.org/globalpublichealth/s/figures**

**https://journals.plos.org/globalpublichealth/s/figures#loc-file-requirements**

**Authors’ response:** We have done so in the revision.

-----

**Additional Editor Comments (if provided):**

**[Note: HTML markup is below. Please do not edit.]**

**Reviewers' comments:**

**Reviewer's Responses to Questions**

**Comments to the Author**

**1. Does this manuscript meet PLOS Global Public Health’s publication criteria? Is the manuscript technically sound, and do the data support the conclusions? The manuscript must describe methodologically and ethically rigorous research with conclusions that are appropriately drawn based on the data presented.**

**Reviewer #1: No**

**Authors’ response:** We appreciate the reviewer’s effort in examining this manuscript and believe we have responded adequately to their detailed comments below.

-----

**2. Has the statistical analysis been performed appropriately and rigorously?**

**Reviewer #1: Yes**

**3. Have the authors made all data underlying the findings in their manuscript fully available (please refer to the Data Availability Statement at the start of the manuscript PDF file)?**

**The PLOS Data policy requires authors to make all data underlying the findings described in their manuscript fully available without restriction, with rare exception. The data should be provided as part of the manuscript or its supporting information, or deposited to a public repository. For example, in addition to summary statistics, the data points behind means, medians and variance measures should be available. If there are restrictions on publicly sharing data—e.g. participant privacy or use of data from a third party—those must be specified.**

**Reviewer #1: Yes**

**4. Is the manuscript presented in an intelligible fashion and written in standard English?**

**PLOS Global Public Health does not copyedit accepted manuscripts, so the language in submitted articles must be clear, correct, and unambiguous. Any typographical or grammatical errors should be corrected at revision, so please note any specific errors here.**

**Reviewer #1: Yes**

**5. Review Comments to the Author**

**Please use the space provided to explain your answers to the questions above. You may also include additional comments for the author, including concerns about dual publication, research ethics, or publication ethics. (Please upload your review as an attachment if it exceeds 20,000 characters)**

**Reviewer #1: 1. Page 4, line 132. “…12-17 years old) stated on July 1, 201, …” . There is a spelling mistake here.**

**Authors’ response:** Thank you for noticing this – it should be 2021, and we made that edit in the manuscript.

-----

**2. Page 5, line 144. “Currently, there is limited research on the decision-making of parents of children under regarding COVID-19 vaccination, particularly in the context of cross-country comparisons “. This sentence is not entirely correct; although there are few studies that make cross-country comparisons, there are many studies on this topic.**

**Authors’ response:** We agree that we could be more specific here. We have changed the last paragraph of the introduction to read (line 140):

“A more global question is what are the patterns of general vaccine hesitancy and acceptance of a COVID-19 vaccine across countries, and do these patterns of vaccine acceptance vary by socioeconomic characteristics. Therefore, the objective of this study is to investigate parental vaccine hesitancy and parents’ acceptance of a COVID-19 for their children in selected countries.”

-----

**3. “Vaccine hesitant” is not defined in the text, but the sources used in the definition are referenced. However, what “vaccine hesitant” means needs to be explained in detail. Otherwise, it is not possible to understand Table 1. For example, in row 1 of Table 1, if the rate of vaccine hesitant is 55%, how can "wants vaccine for self" be 84% and "wants vaccine for child" be 90%?**

**Authors’ response:** We apologize for any confusion. We have separated out these measurements into different paragraphs in the methods (line 164-on), and provided more detail. For vaccine hesitancy, we now have (line 184):

“We also measured general, adult vaccine hesitancy using the validated 10-item adult Vaccine Hesitancy Scale (aVHS) [11]. The aVHS had a 5-point Likert scale as answer choices, ranging from least hesitant (1) to most hesitant (5). Based on a published standard, we dichotomized this variable into vaccine hesitant and non-vaccine hesitant categories [12].”

In this article we separate out general vaccine hesitancy – which represents a psychological state of concerns and indecision (see doi: 10.1016/j.vaccine.2017.08.004) from acceptance of a specific vaccine.

-----

**4. On the right-hand side of Table 1, there are rows whose sum does not add up to 100%: row 5, row 17, row 24, row 28 and row 29.**

**Authors’ response:** Firstly, we slightly edited the first heading row of this table to clarify what we are measuring.

Secondly, it is true that some of the last category (actual vaccination status for self) add to 99% or 101%. We double checked and this is a rounding error (e.g., if the three values were 49.4%, 10.4%, and 40.2%, they would round to 49%, 10%, and 40% and add up to 99%).

-----

**5. How realistic are the definitions used for the vaccine profile (50% effective with a 20% risk of fever, 50% effective with a 5% risk of fever, etc.)? Is there a reference you use when making these definitions? Is fever the only side effect of the vaccine?**

**Authors’ response:** We developed these profiles early in 2020, prior to release of even phase II clinical trial data on preliminary safety and efficacy. The bounds of 50% and 95% refer to something more akin to influenza vaccine effectiveness and measles vaccine effectiveness, respectively. Similarly, one review (doi: 10.1086/422454) found that there was quite a large range of fever after vaccination, with 1%-30% being somewhat common bounds. Our numbers attempted to be best guesses in a situation with little available information. We now detail this in the methods (line 167):

“Before the vaccine was available, we asked about hypothetical acceptance of a COVID-19 vaccine with a given vaccine profile. Individuals were randomized to receive one of four sets of profiles, which differed by safety and effectiveness (50% effective with a 20% risk of fever; 50% effective with a 5% risk of fever; 95% effective with a 20% risk of fever; or 95% effective with a 5% risk of fever). These estimates were chosen for the questionnaire in 2020 and reflect some of the range of vaccine effectiveness seen in other vaccines, with a lower bound from the influenza vaccine [17] and the upper bound from the measles vaccine [18].”

-----

**6. The same findings are repeated on pages 11 and 12.**

**Authors’ response:** We deleted one of the repeats – we appreciate the reviewer’s attention for detail.

-----

**7. What is the reason for including the dates 2021-03 and 2021-06 in the multivariable model in Table 3?**

**Authors’ response:** We have clarified this as “wave of data collection” in the table. Essentially, we believe there could have been a secular trend of changes in vaccine acceptance between the two time points.

-----

**8. Page 13, line 283. “The degree of vaccine preference discordance in children and adults was found to be higher in the United States and Taiwan according to Table 4”. However, In China, the proportion who "want the vaccine for self not child" is 12%, the same as in the US.**

**Authors’ response:** We agree we should be better in our wording on this point. We are now more direct in our wording (line 290):

“The degree of vaccine preference discordance in children and adults is shown in Table 4. Notably, a relatively low proportion in the US and Taiwan wanted a vaccine for themselves and for their children (65% in the US and 62% in Taiwan). In contrast, in other countries, over 80% , of participants expressed willingness for vaccination for themselves and their children.”

-----

**9. Table 4 only includes data for June 2021. What is the reason for this?**

**Authors’ response:** COVID-19 vaccination data, even on intent, is time sensitive, so we chose the last date where we had data from all countries. We have now included this in the methods (line 193):

“we used survey procedures to estimate the frequency of discordant vaccine views between self and child by vaccine hesitancy and education level, separately for each country in the June 2021 wave. We used only one wave out of concern that acceptance and hesitancy regarding COVID-19 vaccines could vary over time.”

-----

**10. Based on the data in Table 1, a discussion comparing vaccination intention or behavior would be more appropriate. The discussion gives the impression that this study only looked at vaccination intentions. However, the authors have data both before and after countries started vaccination. Looking at the data from this perspective makes the article much more valuable.**

**Authors’ response:** We appreciate the reviewer’s suggestion and have done this. For one, we preface part of our methods with this statement (line 165):

“This study includes different vaccine-related measures, including hypothetical acceptance of a vaccine for oneself vs a child, actual vaccination behaviors for oneself, and hesitancy towards vaccines in general – with the perspective of vaccine hesitancy as a psychological state of indecision.”

And in the discussion, we have made edits throughout to highlight our results (see comments below).

-----

**11. In the discussion, topics completely different from the aim of the study and the questions investigated (the relationship of other vaccines such as MMR with sociodemographic factors, etc...) are raised. The discussion should focus on the study data.**

**Authors’ response:** We appreciate the reviewer’s comment. We have substantially revised the discussion. We spent the first few paragraphs explaining our results in the context of the existing literature. We then separate out a section “Implications of research” where we detail a path forward. The discussion now heavily relates back to our aims. (“the objective of this study is to investigate parental vaccine hesitancy and parents’ acceptance of a COVID-19 for their children”)

-----

**12. In its present form, the conclusion is irrelevant to the study data and discussion. The conclusion should be based on the results of the study and make a recommendation based on these data.**

**Authors’ response:** We agree, and our conclusions now more simply read:

“Early in the COVID-19 pandemic, it was already established that in many countries, a substantial proportion of the adult population would refuse a vaccine for themselves. Adolescent COVID-19 vaccination have already been established in many high and middle-income countries, but our study suggests that there is a movement of vaccine hesitancy which could impede the success of future pediatric and adolescent COVID-19 vaccination programs.”

-----
